# The glycoprotein TRP36 of *Ehrlichia* sp. UFMG-EV and related cattle pathogen *Ehrlichia* sp. UFMT-BV evolved from a highly variable clade of *E. canis* under adaptive diversifying selection

**DOI:** 10.1186/s13071-014-0584-5

**Published:** 2014-12-10

**Authors:** Alejandro Cabezas-Cruz, James J Valdés, José de la Fuente

**Affiliations:** Center for Infection and Immunity of Lille (CIIL), INSERM U1019 – CNRS UMR 8204, Université Lille Nord de France, Institut Pasteur de Lille, Lille, France; SaBio, Instituto de Investigación de Recursos Cinegéticos IREC-CSIC-UCLM-JCCM, Ronda de Toledo s/n, 13005 Ciudad Real, Spain; Institute of Parasitology, Biology Centre of the Academy of Sciences of the Czech Republic, České Budějovice, Czech Republic; Department of Veterinary Pathobiology, Center for Veterinary Health Sciences, Oklahoma State University, Stillwatert, OK 74078 USA

**Keywords:** *Ehrlichia* sp. UFMG-EV, *Ehrlichia* sp. UFMT-BV, *E. mineirensis*, Host-shift, Diversifying episodic selection

## Abstract

**Background:**

A new species of *Ehrlichia*, phylogenetically distant from *E. ruminantium*, was found in 2010 infecting cattle in Canada. In 2012 and 2013, we reported the *in vitro* propagation, molecular and ultrastructural characterization of *Ehrlichia* sp. UFMG-EV (*E. mineirensis*), a new species of *Ehrlichia* isolated from the haemolymph of Brazilian *Rhipicephalus (Boophilus) microplus* ticks. A new organism, named *Ehrlichia* sp. UFMT-BV, closely related to *Ehrlichia* sp. UFMG-EV, was recently described in Brazil and after experimental infection it was shown to be pathogenic for cattle. This new emerging clade of cattle *Ehrlichia* pathogens is closely related to *E. canis*. The major immunogenic Tandem Repeat Protein (TRP36; also known as gp36) is extensively used to characterize the genetic diversity of *E. canis*. Homologs of TRP36 were found in both *Ehrlichia* sp. UFMG-EV and *Ehrlichia* sp. UFMT-BV.

**Findings:**

Herein, we characterized the evolution of this new *Ehrlichia* clade using TRP36 sequences. Our working hypothesis is that *Ehrlichia* sp. UFMG-EV and related microorganisms evolved from a highly variable *E. canis* clade. In support of our hypothesis we found that *Ehrlichia* sp. UFMG-EV and *Ehrlichia* sp. UFMT-BV TRP36 evolved from a highly divergent and variable clade within *E. canis* and this clade evolved under episodic diversifying selection with a high proportion of sites under positive selection.

**Conclusion:**

Our results suggest that *Ehrlichia* sp. UFMG-EV and *Ehrlichia* sp. UFMT-BV evolved from a variable clade within *E. canis*.

**Electronic supplementary material:**

The online version of this article (doi:10.1186/s13071-014-0584-5) contains supplementary material, which is available to authorized users.

## Findings

### *Ehrlichia* sp. UFMG-EV and *Ehrlichia* sp. UFMT-BV belong to a new clade of cattle-related Ehrlichia

Anaplasmataceae is a family of α-proteobacteria that includes the genera *Anaplasma*, *Ehrlichia*, *Neorickettsia* and *Wolbachia*. From these genera, *Ehrlichia* and *Anaplasma* are important pathogens affecting animals and humans. *Ehrlichia* are obligate intracellular gram-negative, tick-borne bacteria that grow within membrane-bound vacuoles in human and animal leukocytes causing ehrlichiosis. With a worldwide distribution ehrlichioses are considered emerging diseases that can cause serious illness in a variety of hosts, including humans, livestock and pets. Three news species of cattle-related *Ehrlichia* spp have been recently reported: (i) a new species that naturally infect cattle from British Columbia, Canada [1], (ii) *Ehrlichia* sp. UFMG-EV (referred as *E. mineirensis* in [2,3]) that was isolated from *R. microplus* hemolymph [2-4], and (iii) *Ehrlichia* sp. UFMT-BV that was found to be pathogenic for cattle in Brazil [5]. These three organisms are closely related to *E. canis* [1,2,5]. *Ehrlichia* sp. UFMG-EV and *Ehrlichia* sp. UFMT-BV, however, present new sequence of tandem repeats different to the one reported for *E. canis* TRP36 [2,5,6].

The results of this work expand on our previous findings regarding the evolution and differentiation of TRP36 in *Ehrlichia* sp. UFMG-EV [2]. Herein, we showed that the gene *trp36* presents episodic bursts of selection, unequally distributed across sites and that diversifying selection occurs only in few branches of the *trp36* phylogenetic tree. Our results showed that *Ehrlichia* sp. UFMG-EV and the new *Ehrlichia* sp. UFMT-BV affecting cattle evolved from a highly divergent and variable clade within *E. canis*.

### *Ehrlichia* sp. UFMG-EV *trp36* gene evolved from a highly divergent clade within *E. canis*

**Figure 1:**
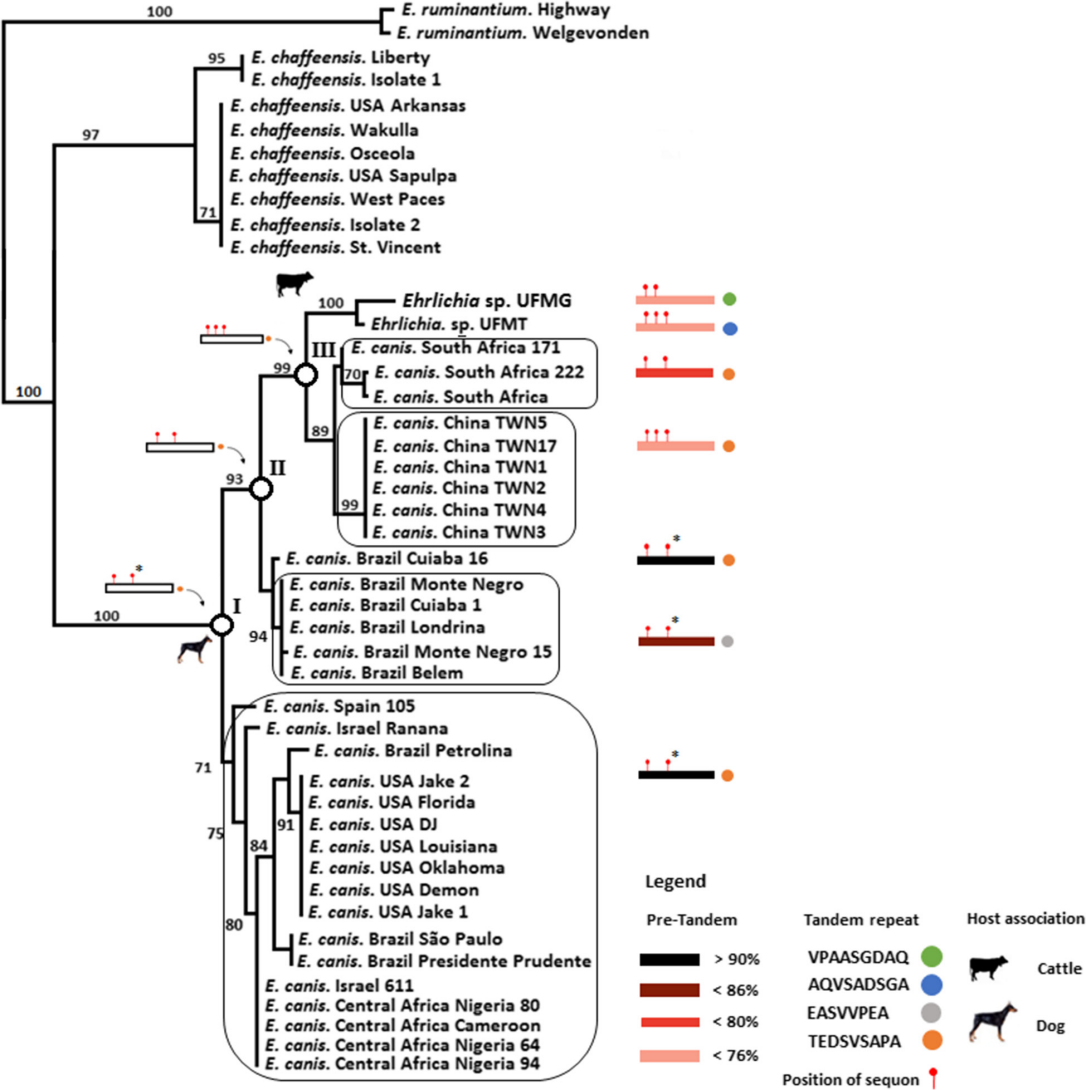
***Ehrlichia*** **sp. UFMG-EV and** ***Ehrlichia*** **sp. UFMT-BV strain belong to a variable clade within** ***E. canis*** **.** The *trp36* (*E. canis*), *gp47* (*E. chaffeensis*) and mucin like protein (*E. ruminantium*) nucleotides sequences were aligned and gap regions removed. Phylogenetic analyses were conducted using ML and NJ. The figure shows that *Ehrlichia* sp. UFMG-EV and *Ehrlichia* sp. UFMT-BV fall in a divergent clade of *E. canis trp36* having low homology (less than 80%: red and pink boxes) compared to the isolate *E. canis* USA Jake 2. The amino acid sequence of the different TRP36 tandem repeats variants are shown (Coloured circles). The positions of the sequons are shown (red sticks on the boxes). The position of TRP36 ancestor clades I, II and III at internal branches (white circles) and position of sequons on ancestors (red sticks on white boxes) are also shown. The topologies obtained with the two methods were similar. The numbers above the internal branches represent bootstrap values. Only bootstrap values higher that 70 are shown.

To study the evolution of *trp36* gene we used a combination of phylogenetic and evolutionary analysis (see Additional file 1 for detailed description of materials and methods). The gene *trp36* has been widely used to study the genetic diversity of *E. canis* strains [7-10]. We performed maximum likelihood and neighbor joining phylogenetic analyses with *trp36* nucleotide sequences available in GenBank (Additional file 1) to study the evolution of *Ehrlichia* sp. UFMG-EV and *Ehrlichia* sp. UFMT-BV *trp36* in relation to *E. canis trp36*. The phylogenetic analysis showed that *Ehrlichia* sp. UFMG-EV and *Ehrlichia* sp. UFMT-BV *trp36* are separated but clustered together with *E. canis* strains from South Africa, Taiwan and Brazil (Figure 1). Using the *E. canis* strain USA Jake-2 as a reference, the TRP36 amino acid sequences from the Taiwanese and South African *E. canis* strains, together with *Ehrlichia* sp. UFMG-EV and *Ehrlichia* sp. UFMT-BV, presented the lowest percent (<86%) of homology (Figure 1, red and pink boxes). The results demonstrated that *E. canis* strain USA Jake-2 belongs to a conservative TRP36 clade within *E. canis* (Figure 1). Members of this clade have a high percent (>90%) of amino acid homology in TRP36 (Figure 1, black boxes).

### The new TRP36 tandem repeat variants evolved from the typical *E. canis* tandem repeat

The tandem repeat composition of the divergent clade was highly variable, encoding the typical *E. canis* TRP36 tandem repeat (TEDSVSAPA), but also other variants – AQVSADSGA (*Ehrlichia* sp. UFMT-BV), EASVVPEA (New Brazilian variant of *E. canis*) and VPAASGDAQ (*Ehrlichia* sp. UFMG-EV) (Figure 1, coloured circles). The conservative TRP36 clade, however, only presented the tandem repeat variant TEDSVSAPA amongst all members. Ancestral sequence reconstruction (see Additional file 1 for detailed description of ancestral sequence reconstruction methods) showed that all the new TRP36 variants evolved from the typical TRP36 tandem repeat, TEDSVSAPA (Figure 1, white circles and roman numerals).

There is currently no experimental evidence that TRP36 has N-linked glycans. The evolution of highly divergent variants of TRP36, however, was associated with an increase in the number of sequons of N-glycosylation in TRP36 (Figure 1, red sticks on colored boxes). In agreement with this finding, the evolution of TRP36 ancestors from clades I to III was associated with the gain of one sequon of N-glycosylation for each evolutionary step (from I to II and from II to III – Figure 1, red sticks on white boxes). One of three sequons present in the ancestor of TRP36 clade III was lost in *Ehrlichia* sp. UFMG-EV and in the South African strains, but it is present in *Ehrlichia* sp. UFMT-BV and the Taiwanese strains. The second sequon in TRP36 ancestor clade I and the strains from USA, Spain, Israel, Central Africa and Brazil possess a proline (P) residue in the second position making it improbable that the asparagine (N) will be glycosylated (Figure 1, asterisks on red sticks). The relevancy of whether these sequons are glycosylated or not is that changes in glycosylation patterns may contribute to evade host immune system [11] and antigenic drift [12].

### *Ehrlichia* sp. UFMG-EV *trp36* evolved under episodic diversifying selection

**Figure 2:**
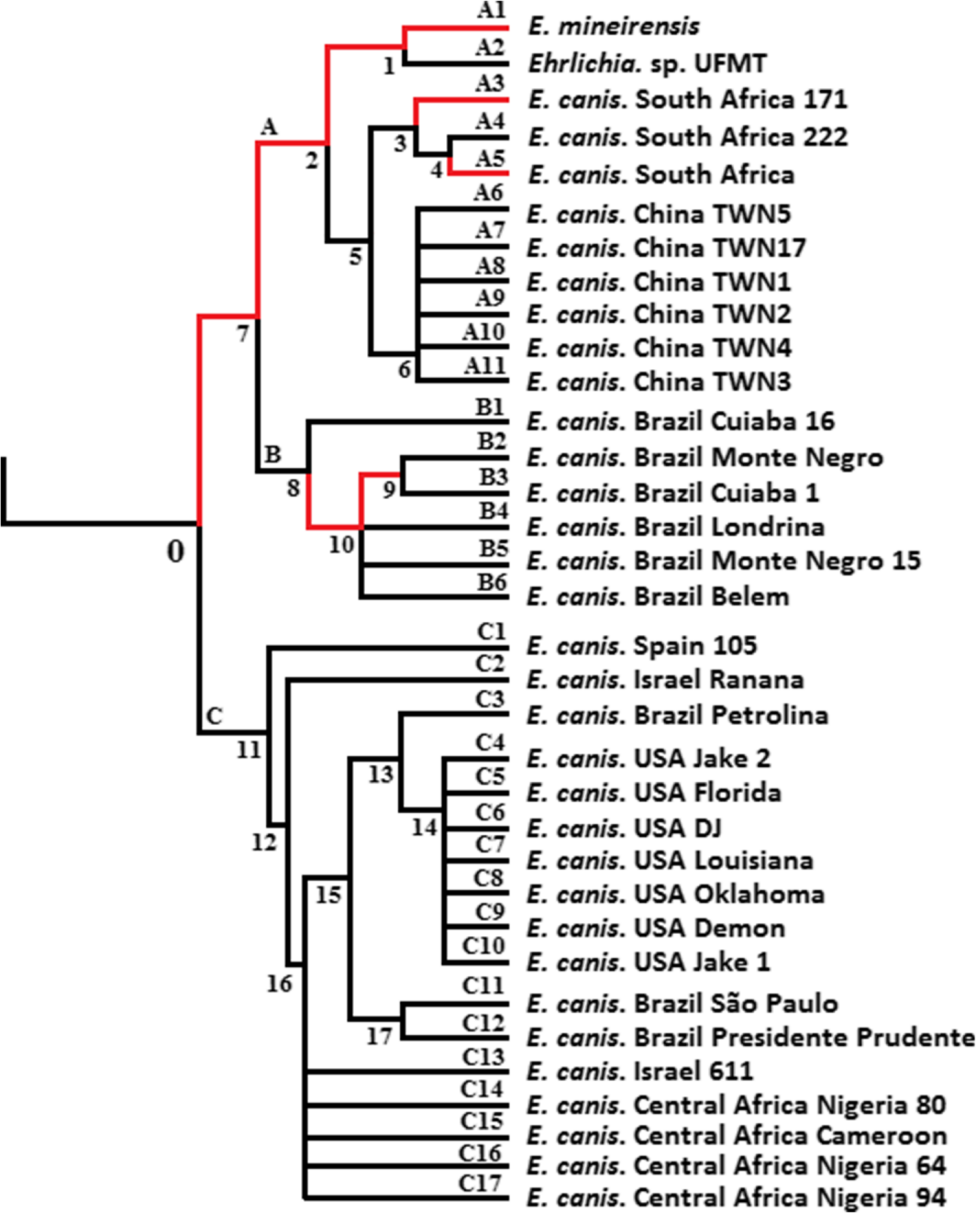
**Branches under episodic diversifying selection in the** ***trp36*** **tree.** The tree of *trp36* orthologs is shown. Branch-site REL model (Additional file 1) was used to determine branches under episodic diversifying selection (highlighted in red). Branches were considered under episodic diversifying selection when corrected *p-value* < 0.05 (see Additional file 1 for methods). For the rest of the tree (branches in black) there is no evidence of episodic diversifying selection (Additional file 2).

Our next step was to test whether different branches or codon sites of the *trp36* phylogenetic tree evolved under episodic diversifying selection. Results showed that the diversifying selection events among the branches were scarce along the phylogenetic tree (Figure 2). Only 8 (A1, A3, A5, 1, 2, 7, 9 and 10) out of 51 (15.6%) branches were found to be under episodic diversifying selection (Corrected *p-value* ≤ 0.05 – Figure 2 and Additional file 2). Episodic diversifying selection was detected only in branches belonging to the highly divergent clade of TRP36 described above (Figure 1). The patterns of episodic diversifying selection were complex, with differences in extent and strength of selection along the diversifying branches. The branches can be separated into four groups: (i) 2, 9, A1, A3 and A5 that experienced strong selective force (ω+ > 3333.56) in a small proportion of sites (Proportion < 0.07), (ii) 1 that experienced low selective force (ω + = 7.86) in a high proportion of sites (Proportion = 0.17), (iii) 7 that experienced low selective force (ω + = 46.08) in a low proportion of sites (Proportion = 0.05), and (iv) 10 that experienced middle selective force (ω + = 166.14) in a high proportion of sites (Proportion = 0.15). Among the branches experiencing episodic selection, 11 out of 171 (6.4%) codon sites were under episodic diversifying selection (Table 1, Additional file 3). Most of these sites were concentrated in branches 7 and 1.

**Table 1:** **Codons under episodic diversifying selection in specific branches**

**Codons**	**Branches** ^**a**^	**From**	**To**	**Type of substitution** ^**b**^
10	7	aac (N)	ggt (G)	dN and dS
21	7	caa (Q)	tca (S)	dN
28	7	tca (S)	gta (V)	dN
	1	gta (V)	aca (T)	dN
39	7	cat (H)	agt (S)	dN
	1	agt (S)	cat (H)	dN
40	7	cct (P)	ggt (G)	dN
51	6	aat (N)	ggt (G)	dN
77	15	gct (A)	gtt (V)	dN
	B1	gct (A)	gtt (V)	dN
105	2	tat (Y)	gaa (E)	dN
124	5	aat (N)	tct (S)	dN
142	8	tct (S)	ggt (G)	dN
	10	tct (S)	gaa (E)	dN and dS
145	1	gct (A)	gtt (V)	dN
	A1	gtt (V)	caa (Q)	dN and dS

^a^Internal and external branches are identified by numbers and letters and numbers as in Figure 2.

^b^Type of substitution: non-synonymous (dN) and synonymous (dS).

Searching the sequences for evidence of positive and negative selection using SLAC, FEL, REL and MEME (see materials and methods) showed that many sites experienced positive or negative selection (Table 2). The higher proportion of sites inferred to be evolving under positive selection was found in the ancestral branches 1, 7 and 10. The branches A1, A2, B2-B6, which are associated to deep branches 1 and 10 (Figure 2), were related to the occurrence of new forms of TRP36 tandem repeats (Figure 1). This relation suggests that early, strong selective events on lineages 1 and 10 may have been related to the occurrence of new tandem repeats. The sites under negative selection were concentrated in ancestral lineage 2.

**Table 2:** **Codons under positive and negative selection**

**Codons**	**Branches** ^**a**^	**SLAC ω** ^**b**^	**SLAC p-value**	**FEL ω** ^**b**^	**FEL p-value**	**REL ω** ^**b**^	**Bayes factor**	**MEME ω** ^**b**^	**MEME p-value**	**Selection type** ^**c**^
6	B5	−4.047	0.333	−3.159	0.081	−1.126	229.790	-	-	Negative
8	7	−4.604	0.293	−4.597	0.071	−1.096	92.479	-	-	Negative
10	7	−0.686	0.787	−2.465	0.583	−0.149	0.595	>100	0.030	Positive
21	7	4.991	0.423	3.013	0.222	0.810	3.221	>100	0.019	Positive
28	7	8.543	0.184	5.818	0.082	1.678	28.536	>100	0.010	Positive
	1									
39	7	6.912	0.371	3.907	0.444	1.265	8.352	>100	0.013	Positive
	1									
40	7	4.047	0.444	2.432	0.350	0.476	1.985	>100	0.011	Positive
49	1	−16.858	0.031	−10.024	0.007	−1.120	178.481	-	-	Negative
	6									
51	6	3.473	0.603	1.923	0.586	0.096	1.136	>100	0.028	Positive
52	1	−8.094	0.111	−3.234	0.062	−1.087	77.050	-	-	Negative
	6									
71	1	−18.415	0.026	−14.187	0.004	−1.121	187.132	-	-	Negative
	2									
77	B1	4.047	0.444	2.150	0.377	0.340	1.681	>100	0.025	Positive
	15									
83	5	−4.047	0.333	−3.402	0.073	−1.127	243.194	-	-	Negative
89	2	−9.551	0.153	−7.402	0.042	−1.101	99.411	-	-	Negative
94	2	−6.708	0.201	−4.324	0.059	−1.124	189.451	-	-	Negative
96	2	−4.047	0.333	−4.119	0.063	−1.127	242.661	-	-	Negative
105	2	3.569	0.689	2.357	0.608	0.330	1.536	>100	0.030	Positive
116	2	−7.488	0.180	−3.380	0.074	−1.126	199.960	-	-	Negative
124	5	3.663	0.543	2.278	0.488	0.329	1.569	>100	0.004	Positive
139	10	−17.080	0.037	−7.190	0.074	−1.007	23.196	-	-	Negative
142	1	6.862	0.316	6.098	0.511	1.459	31.512	>100	0.009	Positive
	10									
145	1	4.902	0.413	4.701	0.571	1.230	7.496	>100	0.017	Positive
	A1									
152	10	20.127	0.244	41.463	0.238	1.510	66.006	>100	0.224	Positive
	B4									
	A5									
158	1	2.355	0.585	−1.195	0.886	1.162	161.941	1.219	0.612	Positive
	4									
	A4									
	A5									
	10									
162	1	16.368	0.227	8.312	0.354	1.574	87.208	>100	0.343	Positive
	5									
	10									
	B2									
166	1	25.338	0.042	5.483	0.239	1.510	20.855	>100	0.198	Positive
	10									
	A8									
167	10	28.242	0.032	8.115	0.083	1.677	53.176	>100	0.084	Positive
	B1									
170	10	−8.379	0.406	−74.785	0.031	0.536	0.396	-	-	Negative
	9									

^a^Internal and external branches are identified by numbers and letters and numbers as in Figure 2.

^b^The ratio between non-synonymous (dN) and synonymous (dS) nucleotide substitution per site (**ω**) analyzed by Datamonkey via SLAC, FEL, REL and MEME.

^c^Sites were considered under positive selection (**ω > 1**) or negative selection (**ω < 1**) when at least one of the methods shows significant difference (*p-value* < 0.05 (SLAC, FEL and MEME) or Bayes Factor > 50 (REL)).

Codon 77 evolved under diversifying (positive) and codon 116 evolved under negative selection. These two codons code for amino acids involved in the formation of sequons among TRP36 homologs (Additional file 4). While codon 77 was selected in branches 15 and B1 (*E. canis*), codon 116 was selected in branch 2 (*E. canis*, *Ehrlichia* sp. UFMT-BV and *Ehrlichia* sp. UFMG-EV). This data therefore suggests that putative N-glycosylation associated with this sequon might be important in the host shift (see below) observed in *Ehrlichia* sp. UFMT-BV and *Ehrlichia* sp. UFMG-EV.

### Model of emergence of *Ehrlichia* sp. UFMG-EV and *Ehrlichia* sp. UFMT-BV within *E. canis*

The emergence of new pathogens is frequently associated to mutations that confer the ability to infect novel hosts, known as “host shift” [13]. *Ehrlichia* sp. UFMG-EV and *Ehrlichia* sp. UFMT-BV are closely related to *E. canis*, however they were associated to new invertebrate and vertebrate hosts, respectively. First, while the common tick vector for *E. canis* is *R. sanguineus* [14], *Ehrlichia* sp. UFMG-EV was isolated from *R. microplus* hemolymph [2]. Secondly, while *E. canis* is mainly pathogenic for dog [10], *Ehrlichia* sp. UFMT-BV was found to be pathogenic for cattle [5]. How pathogens can colonize new hosts is a challenging question in evolutionary biology [13]. Recently, Aguiar and colleagues [9] suggested that *E. canis* may have a wider range of hosts in Brazil than currently recognized. The host shift in this context may have occurred in a scenario where dogs infected with a variable *E. canis* strain, as previously found in Brazil [9], were the source of infection for *R. microplus* or *R. sanguineus* ticks that later infested cattle. Both tick species are able to infect dogs [15,16] and cattle [17]. The scenario involving *R. microplus* is unlikely as this is a one-host tick species. However, *R. microplus* moves among hosts during their parasitic lifetime [18], thereby increasing the chances of horizontal pathogen transmission among different hosts. Changes in evolutionary pressures on *E. canis*, related to new host association, may have resulted in a completely new species.

Our evidence supports the idea of differential evolutionary pressures on the glycoprotein TRP36 along different strains of *E. canis*, resulting in highly divergent variants of TRP36. In the habitual host of *E. canis*, TRP36 must possess amino acid positions beneficial or neutral that may be deleterious in new hosts – the opposite may also be true. Within variable strains of a given pathogen, novel genetic variants may eventually deliver beneficial mutations that promote successful emergence, thereby providing a source for adaptive genetic variation in new hosts [13]. In agreement with this, we found a large proportion of sites that evolved under purifying (negative) selection, positive and diversifying selection. It is worth noting that the selective events were more frequent and strong in the deepest branches of *trp36* phylogenetic tree. This supports the hypothesis that most mutations that originated in the new TRP36 amino acid variants of *Ehrlichia* sp. UFMG-EV and *Ehrlichia* sp. UFMT-BV occurred before the emergence of the clade formed by these two organisms. The fact that the most recent common ancestor (Figure 1, ancestor clade III) between *Ehrlichia* sp. UFMG-EV, *Ehrlichia* sp. UFMT-BV and *E. canis* had a typical TRP36 tandem repeat structure, supports the aforementioned hypothesis. The divergence found in TRP36 tandem repeats was consistent with a 1.7% sequence divergence between *16SrRNA* of *Ehrlichia* sp. UFMG-EV and *E. canis* [2]. Taking into account the high identity of *16SrRNA* among *E. canis* strains (maximum 0.6%) [7], and thus the conservative nature of this gene, *Ehrlichia* sp. UFMG-EV may have diverged a long time ago from *E. canis*.

## Conclusion

Altogether, these results suggest that this new group of organisms evolved from *E. canis* sensu stricto and has become ecologically independent from the parental species. In agreement with the new hosts association of this group of microorganisms, it was found that *Ehrlichia* sp. UFMG-EV was able to propagate in bovine aorta BA886 cell line, while *E. canis* did not [4]. This *in vitro* observation supports the above conclusions regarding the new host specificity of this novel group of cattle related agents. At the ultrastructural level, *Ehrlichia* sp. UFMG-EV shares ultrastructural features with other members of the genus *Ehrlichia* (*E. muris*, *E. canis* and *E. chaffeensis*). We found cells, however, with unusual structures (invagination of the cellular membrane) for which we yet do not have an explanation [3]. Further studies should clarify the role of major immunogenic surface exposed proteins in the evolution of bacterial host shift. The full genome of *E. mineirensis* (*Ehrlichia* sp. UFMG-EV) might be an important contribution to these studies.

## Additional files

Additional file 1:
**Detailed description of materials and methods.**
Additional file 2:
**Likelihood ratio test statistics for branches under episodic diversifying selection.** Branch-site REL model (Additional file 1) was used to determine branches under episodic diversifying selection (highlighted in red). Branches were considered under episodic diversifying selection when corrected *p-value* < 0.05 (see Additional file 1 for methods). Additional file 3:
**Likelihood ratio test statistics for sites under episodic diversifying selection.** MEME (Additional file 1) was used to determine sites (codons) under episodic diversifying selection (highlighted in red). Sites were considered under episodic diversifying selection when *p-value* < 0.05 (see Additional file 1 for methods). Additional file 4:
**Sequons of N-glycosylation in the N-terminus of TRP36 variants.** Putative sequons of N-glycosilation of the N-terminus of TRP36 variant included in this study are shown.
