# Supplementary material for: The glycoprotein TRP36 of Ehrlichia sp. UFMG-EV and related cattle pathogen Ehrlichia sp. UFMT-BV evolved from a highly variable clade of E. canis under adaptive diversifying selection
Source: Parasit Vectors. 2014 Dec 10;7:584. doi: 10.1186/s13071-014-0584-5 (PMC4266974; doi:10.1186/s13071-014-0584-5)
Supplement: Additional file 1: — Detailed description of materials and methods. [file 13071_2014_584_MOESM1_ESM.pdf]

**Detailed description of materials and methods**

**Phylogenetic trees**

The phylogenetic analysis were conducted with *gp36* nucleotide sequences aligned with MAFFT (v7) configured for the highest accuracy [1]. After alignment, regions with gaps were removed from the alignment using Gblocks (v0.91b) [2]. Phylogenetic trees were reconstructed using maximum likelihood (ML) and neighbor joining (NJ) methods as implemented in PhyML (v3.0 aLRT) [3,4] and PHYLIP (v3.66) [5], respectively. The reliability for the internal branches of ML was assessed using the bootstrapping method (1000 bootstrap replicates) and the approximate likelihood ratio test (aLRT – SH-Like) [3]. Reliability for the NJ tree was assessed using bootstrapping method (1000 bootstrap replicates). The GenBank accession numbers of the *gp36* sequences used in the phylogenetic analyses are as follow, *E. mineirensis*: AFV15304; *Ehrlichia* sp. UFMT: AHI42992; *E. canis* USA-Louisiana: ABA39254; *E. canis* Brazil-Sao_Paulo: ABA39257; *E. canis* Central Africa-Cameroon: ABA39258; *E. canis* Central Africa-Nigeria 64: JN622143; *E. canis* Central Africa-Nigeria 94: JN982341; *E. canis* Central Africa-Nigeria 80: JN982338; *E. canis* South Africa-222: KC479021; *E. canis* Spain-105: KC479019; *E. canis* South Africa-171: KC479020; *E. canis* Israel-611: ABV02078; *E. canis* Israel-Ranana: ABW91006; *E. canis* China-TWN1: ABS82573; *E. canis* China-TWN2: ABU44524; *E. canis* China-TWN3: ABV26011; *E. canis* China-TWN4: ABX71625; *E. canis* China-TWN5: ADJ68334; *E. canis* China-TWN17: ADL27830; *E. canis* USA-Oklahoma: AAZ40200; *E. canis* USA-Florida: ABA39255; *E. canis* USA-Jake-2: AAZ68160; *E. canis* USA-DJ: ABA39256; *E. canis* USA-Jake-1: AAZ40199; *E. canis* USA-Demon: AAZ40201; *E. canis* South African: AGS13119; *E. canis* Brazil Petrolina Northeast: AFS49961; *E. canis* Brazil Londrina Southern: AFS49963; *E. canis* Brazil Cuiaba 1 Midwest: AFS49962; *E. canis* Brazil Monte Negro 24

Northern: AFS49965; *E. canis* Brazil Belem Northern: AFT92033; *E. canis* Brazil Monte Negro 15 Northern: AFS49964; *E. canis* Brazil Cuiaba16 Midwest: AFS49960; *E. canis* Brazil Presidente Prudente Southeast: AFS49959; *E. chaffeensis* USA-Arkansas: AAZ40202; *E. chaffeensis* USA-Sapulpa: AAZ40203; *E. chaffeensis* Wakulla: AHX09877; *E. chaffeensis* Osceola: AHX07953; *E. chaffeensis* St.Vincent: AHX08591; *E. chaffeensis* West Paces: AHX10533; *E. chaffeensis* Liberty: AHX06964; *E. chaffeensis* isolate 1: ABA39261; *E. chaffeensis* isolate 1: ABA39259; *E. ruminantium* Highway: AF308673; *E. ruminantium*: WP 011256013 and *E. ruminantium* Welgevonden: WP 011154795.

**Evolutionary analyses**

Codon based alignment was performed using the codon suite server [6,7]. All the evolutionary analyses were performed using the Datamonkey webserver (http://www.datamonkey.org; [8]). Detection of selection pressure on individual codons were calculated using four methods, single likelihood ancestor counting (SLAC) [9], fixed effects likelihood (FEL) [9], random effects likelihood (REL) [10], and mixed-effects model of evolution (MEME) [11]. Positive and negative selection were assigned to codons where ω=dN (non-synonymous substitutions)/dS (synonymous substitutions) ratio was higher or lower than 1 respectively. Sites were considered under positive selection (ω > 1) or negative selection (ω < 1) when at least one of the methods shows significant difference (*p-value* < 0.05 (SLAC, FEL and MEME) or Bayes Factor > 50 (REL)). Episodic diversifying selection reflects adaptive evolution. The episodic burst of selection is unequally distributed across sites in a gene, and only occurs to few lineages in a phylogenetic tree. To identify branches under episodic diversifying selection in the *gp36* phylogenetic tree, we use the unrestricted Branch-site REL model [12]. In this method, substitution rates are allowed to vary from branch to branch and from site to site in a random fashion where unobserved strengths of selection at sites and

branches are incorporated using a probability distribution. The parameters of this distribution are estimated using a maximum likelihood criteria [12], REL. Afterwards, we determined the specific codon sites under episodic diversifying selection using MEME [11]. The MEME method uses a fixed effect for codon sites while randomizing effects for branches where the ratio of non-synonymous and synonymous substitution sites are allowed to vary along branches according to their distribution, as positively (ratio > 1) or negatively selected (ratio < 1).

Ancestral amino acid sequences were determined for three groups of sequences, using the substitution model better fitting the actual data (CodonTest; [13]): (i) South African, Taiwanese, E. mineirensis, Ehrlichia sp. (UFMT-BV strain) and Brazilian strains, using the VT (Vingron and Muller) + F model; (ii) All gp36 amino acid sequences using the JTT (Jones,Taylor,Thornton) + F model and (iii) South African, Taiwanese, E. mineirensis, Ehrlichia sp. (UFMT-BV strain) using Dayhoff model. The reconstruction of the ancestral amino acid sequence was performed using a Neighbour Joining Tree under the respective model of substitutions. Three reconstruction methods were used: Joint [14], marginal [15] and sample [16].

## References


1. Katoh K, Standley DM: **MAFFT multiple sequence alignment software version 7: improvements in performance and usability.** *Mol Biol Evol* 2013, **30:**772-780.

2. Castresana J: **Selection of conserved blocks from multiple alignments for their use in phylogenetic analysis.** *Mol Biol Evol* 2000, **17:**540-552.

3. Anisimova M, Gascuel O: **Approximate likelihood-ratio test for branches: A fast, accurate, and powerful alternative.** *Syst Biol* 2006, **55:**539-552.

4. Guindon S, Gascuel O: **A simple, fast, and accurate algorithm to estimate large phylogenies by maximum likelihood.** *Syst Biol* 2003, **52:**696-704.

5. Felsenstein J: **PHYLIP - Phylogeny Inference Package (Version 3.2).** *Cladistics* 1989, **5:**164-166.

6. Schneider A, Cannarozzi G, Gonnet G: **Empirical codon substitution matrix.** *BMC Bioinformatics* 2005, **6:**134.

7. Schneider A, Gonnet G, Cannarozzi G: **SynPAM-a distance measure based on synonymous codon substitutions.** *IEEE/ACM Trans Comput Biol Bioinform* 2007, **4:**553-560.

8. Delport W, Poon A, Frost S, Kosakovsky P: **Datamonkey 2010: a suite of phylogenetic analysis tools for evolutionary biology.** *Bioinformatics* 2010, **26:**2455-2457.

9. Pond SL, Frost SD: **Datamonkey: rapid detection of selective pressure on individual sites of codon alignments.** *Bioinformatics* 2005, **21:**2531-2533.

10. Yang Z: **Maximum likelihood phylogenetic estimation from DNA sequences with variable rates over sites: approximate methods.** *J Mol Evol* 1994, **39:**306-314.

11. Murrell B, Wertheim JO, Moola S, Weighill T, Scheffler K, Kosakovsky PSL: Detecting Individual Sites Subject to Episodic Diversifying Selection. *PLoS Genet* 2012, **8:**e1002764.

12. Kosakovsky Pond SL, Murrell B, Fourment M, Frost SD, Delport W, Scheffler K: **A random effects branch-site model for detecting episodic diversifying selection.** *Mol Biol Evol* 2011, **28:**3033-3043.

13. Delport W, Scheffler K, Botha G, Gravenor MB, Muse SV, Kosakovsky PSL: **CodonTest: modeling amino acid substitution preferences in coding sequences.** *PLoS Comput Biol* 2010, **6:**e1000885

14. Pupko T, Shamir IPR, Graur D: **A fast algorithm for joint reconstruction of ancestral amino acid sequences.** *Mol Biol Evol* 2000, **17:**890-896

15. Yang Z, Kumar S, Nei M: **A new method of inference of ancestral nucleotide and amino acid sequences.** *Genetics* 1995, **14:**1641-1650.

16. Nielsen R: **Mapping mutations on phylogenies.** *Syst Bio* 2002, **51:**729-739.
