# Supplementary material for: The glycoprotein TRP36 of Ehrlichia sp. UFMG-EV and related cattle pathogen Ehrlichia sp. UFMT-BV evolved from a highly variable clade of E. canis under adaptive diversifying selection
Source: Parasit Vectors. 2014 Dec 10;7:584. doi: 10.1186/s13071-014-0584-5 (PMC4266974; doi:10.1186/s13071-014-0584-5)
Supplement: Additional file 4: — Sequons of N-glycosylation in the N-terminus of TRP36 variants. Putative sequons of N-glycosilation of the N-terminus of TRP36 variant included in this study are shown. [file 13071_2014_584_MOESM4_ESM.pdf]

N-glycosylation sites in the N-terminus of TRP36 variants.

Strains	Position of the Asparagine (N)	Sequon
Ehrlichia sp. UFMG-EV	72	NRSL
	97	NFSI
Ehrlichia sp. UFMT-BV	76	NASF
	97	NFSI
	115	NASF
E. canis South Africa 171	76	NASF
	115	NSSL
E. canis South Africa 222	76	NASF
	115	NSSL
E. canis South African	76	NASF
	115	NSSL
E. canis China TWN1	76	NASF
	97	NFSI
	115	NSSL
E. canis China TWN2	76	NASF
	97	NFSI
	115	NSSL
E. canis China TWN3	76	NASF
	97	NFSI
	115	NSSL
E. canis China TWN4	76	NASF
	97	NFSI
	115	NSSL
E. canis Brazil Cuiaba 16	76	NVSF
	116	N**P**SR
E. canis Brazil Monte Negro	76	NASF
	116	N**P**SR
E. canis Brazil Cuiaba 1	76	NASF
	116	N**P**SR
E. canis Brazil Londrina	76	NASF
	116	N**P**SR
E. canis Brazil Monte Negro 15	76	NASF
	116	N**P**SR
E. canis Brazil Belem	76	NASF
	116	N**P**SR
E. canis Spain-105	76	NASF
	116	N**P**SR
E. canis Israel Ranana	76	NASF
	116	N**P**SR
E. canis Brazil Petrolina	76	NVSF
	116	N**P**SR
E.canis.USA-Jake-2	76	NVSF
	116	N**P**SR
E. canis USA Florida	76	NVSF
	116	N**P**SR
E. canis USA-DJ	76	NVSF

	116	N**P**SR
E. canis USA-Louisiana	76	NVSF
	116	N**P**SR
E. canis USA Oklahoma	76	NVSF
	116	N**P**SR
E. canis USA Demon	76	NVSF
	116	N**P**SR
E. canis USA Jake-1	76	NVSF
	116	N**P**SR
E. canis Brazil São Paulo	76	NVSF
	116	N**P**SR
E. canis Brazil Presidente Prudente	76	NVSF
	116	N**P**SR
E. canis Israel 611	76	NASF
	116	N**P**SR
E. canis Central_Africa Nigeria 80	76	NASF
	116	N**P**SR
E. canis Central Africa-Cameroon	76	NASF
	116	N**P**SR
E. canis Central Africa Nigeria 64	76	NASF
	116	N**P**SR
E. canis Central. Africa Nigeria 94	76	NASF
	116	N**P**SR
